# Hybrid Work Profiles and Team Functioning: A Person-Centered Analysis of Hybrid Work Quality

**DOI:** 10.17505/jpor.2026.29456

**Published:** 2026-08-24

**Authors:** Simona Margheritti, Simone Donati, Andrea Gragnano, Salvatore Zappalà, Massimo Miglioretti

**Affiliations:** 1 University of Milano-Bicocca, Department of Psychology; 2 University of Bologna, Department of Psychology

**Keywords:** Hybrid Work, Hybrid Teams, Telework Quality, Team Functioning, Latent Profile Analysis, Work and Organizational Psychology, Person-Centered Approach

## Abstract

Hybrid work has become a widespread organizational practice, yet employees’ experiences remain highly heterogeneous. Adopting a person-oriented perspective, this study examines whether employees’ perceptions of hybrid work resources are associated with distinct patterns of team-related processes. Drawing on the Quality of Telework (QoT) framework, hybrid work quality is conceptualized as a configurational phenomenon in which multiple resources co-occur within individuals rather than operating independently. Using latent profile analysis on data from 719 hybrid workers across four organizations, we identified four distinct profiles based on key QoT resources (i.e., remote and office-based workstations, flextime, and enabling management). These profiles differed systematically in team identification, knowledge sharing, collective self-efficacy, satisfaction with hybrid teamwork, and perceived team efficiency. The findings suggest that hybrid work experiences form qualitatively distinct patterns, with more balanced configurations of resources associated with more positive perceptions of team functioning. By focusing on configurations rather than isolated predictors, this study contributes to extending the QoT framework and demonstrates the value of person-oriented approaches for capturing heterogeneity in contemporary work contexts. More broadly, the results highlight how combinations of work-related conditions are linked to different experiential patterns, offering a more nuanced understanding of hybrid work and its implications for team-related processes.

## Introduction

Over the last decade, and even more markedly following the COVID-19 pandemic, remote working has become a widespread and structural feature of contemporary organisations. In the post-pandemic phase, remote work has evolved into hybrid work, in which employees alternate between on-site and remote work, reshaping how work is organised, coordinated, and experienced. This transformation challenges traditional assumptions about time, place, supervision, and collaboration, and places greater emphasis on goal achievement, autonomy, and trust (Grant & Russell, 2020). In this paper, we therefore refer to hybrid work as a mode of working that involves an alternation, on a weekly and monthly basis, between days worked at the company’s premises and days worked from home or other locations outside the company (e.g., co-working spaces).

Research increasingly suggests that remote work is not inherently beneficial or sustainable. Rather, its value depends on how it is implemented and supported within organisations. Miglioretti et al. (2021, 2022) highlighted that not all forms of hybrid work are valuable and that substantial heterogeneity exists in employees’ experiences. This heterogeneity results from the availability of specific organisational resources that enable effective remote work; such resources include the quality of remote workstations, office flexibility, flex-time, and enabling management.

To date, much of the empirical research on hybrid working has focused on individual-level outcomes, such as job satisfaction, well-being, performance (Ahmadi et al., 2022; Allen et al., 2015; Karácsony, 2021; Margheritti et al., 2023; Petcu et al., 2021; Toscano et al., 2025), turnover intention, and role stress (Gajendran & Harrison, 2007).

While this literature has provided important insights, it offers only a partial understanding of the implications of remote and hybrid work in contemporary organisations. The diffusion of remote and hybrid work arrangements has in fact altered the nature of teamwork, as employees increasingly collaborate with colleagues who are geographically dispersed, temporally asynchronous, and connected primarily through digital technologies. As a result, understanding hybrid team functioning has become a critical issue in the study of remote work. Hybrid teams are increasingly characterised by virtuality and distributivity, which distinguish them from traditional face-to-face groups and pose specific challenges for coordination and performance (Bell & Kozlowski, 2002; Henry et al., 2021). These features are shaped by task interdependence, the nature of the work, the supporting technological environment, and temporal distance among tasks, all of which influence how team members interact and coordinate across time and space. In such contexts, implementing high-quality hybrid work is a key strategy for enhancing effectiveness.

Starting from these premises and adopting a person-centred approach, the present study firstly aims to identify the presence of different profiles of hybrid workers, defined on the basis of the resources of the Quality of Telework (QoT) model (Margheritti et al., 2023; Miglioretti et al., 2021). Secondly, this research examines whether the profiles are associated with different employees’ perceptions of hybrid work team functioning. Indeed, from our perspective, the core resources of the QoT Model are likely to be associated not only individual positive outcomes (such as well-being and job satisfaction) but also collective effectiveness in hybrid work settings, where the quality of physical, temporal, and managerial resources is critical for sustaining coordination, collaboration, and shared goals in contexts characterised by high interdependence and reduced co-presence. By examining the profile characteristics that emerged from the latent profile analysis (LPA) of the QoT model’s resources, this study identifies differences in the quality of hybrid work implementation, highlighting implications for both theory and practice. Adopting a person-oriented perspective, the study moves beyond variable-centered approaches to capture how multiple resources co-occur within individuals resulting in distinct configurations of perceived hybrid work experiences. This approach is particularly suited to capturing the complexity of hybrid work contexts and allows for a more nuanced understanding of how different resource combinations are associated with qualitatively distinct patterns of team functioning.

### Theoretical Background and Research Questions

#### The Telework Quality Model

Until Covid-19 pandemic, the work practice in which members of an organization substitute a portion of their typical work hours (from a few hours per week to nearly fulltime) to work away from a central workplace - typically principally from home - using technology to interact with others as needed to conduct work tasks, was usually defined as telework (Allen et al., 2015). In recent years, the literature has emphasized that telework cannot be regarded as inherently beneficial, as its effects on positive individual and organizational outcomes largely depend on the quality of the conditions under which it is implemented (Miglioretti et al., 2021). From this perspective, Miglioretti and colleagues (Miglioretti et al., 2021, 2022), also drawing on the Effort– Recovery approach (Meijman & Mulder, 1998), recently proposed the Telework Quality Model (TQM), which is based on the assumption that high-quality telework requires the simultaneous presence of specific key resources capable of sustaining employees’ investment of energy, skills, and motivation, even when working remotely. The model identifies four core resources. The core resources are (a) agile offices within organizations, defined as flexible and diversified physical work environments that foster interaction, collaboration, and social exchange (e.g., infor-mal meeting rooms, collaborative spaces, shared desk spaces, break rooms, relaxation areas); (b) functional remote workstations, referring to the availability of adequate and well-equipped spaces to work effectively outside the traditional office, primarily at home. In a functional remote workstation, the worker has the required technological equipment and an environmentally suitable workspace (ergonomic, noise-free, and healthy); (c) flex-time, that is, the degree of autonomy granted to employees in managing working hours and schedules; and (d) enabling management, referring to managerial practices that support effective hybrid work by integrating clarity of objectives and transformational leadership. Role clarity ensures that employees understand their responsibilities and what is expected of them, while transformational leadership provides direction, shared goals, and motivational support, fostering engagement and performance in telework contexts.

Taken together, these resources outline a conception of telework that goes beyond minimal technological requirements, instead emphasizing autonomy, flexibility, and accountability, elements closely linked to a culture of trust and empowerment in work management (Martínez-Sánchez et al., 2006; Pigini & Staffolani, 2019) and to a reconsideration of traditional managerial models (Contreras et al., 2020; Donald, 2002; Leede & Kraijenbrink, 2014; Miglioretti et al., 2022). In line with a person-oriented perspective, the Quality of Telework model can be interpreted as a configurational framework, in which multiple resources are expected to cooccur within individuals, forming distinct patterns rather than operating independently. Building on this view, the present study adopts a person-centered approach to conceptualize hybrid work quality as a configurational phenomenon, whereby the four core resources combine to form distinct profiles of hybrid work experience.

Importantly, prior research by Miglioretti et al. (2022) has already applied this perspective and identified four distinct telework quality profiles, reflecting different combinations of the four core resources, but this previous research was conducted during Covid-19 pandemic. These findings provide initial evidence that hybrid work quality is not a matter of isolated resources but rather emerges from meaningful patterns of their joint availability.

Drawing on this evidence, the present study aims to examine the stability of hybrid work quality profiles in the post-pandemic context of hybrid work. Specifically, we expect to identify the same profile patterns previously identified by Miglioretti et al. (2022) in the telework context: (a) a profile characterized by overall poor hybrid work quality, with low levels across all four resources; (b) a profile reflecting overall high hybrid work quality, with high levels across all hybrid work quality dimensions; and (c–d) two intermediate profiles, characterized by high or low levels on specific hybrid work quality resources.

Accordingly, the following hypothesis is proposed:

**Hypothesis 1.** *Four distinct hybrid work quality profiles will emerge, displaying patterns of hybrid work quality resources comparable to those identified by Miglioretti et al. (2022)*.

#### Hybrid team processes and quality of hybrid work

This study assumes that if different workers experience hybrid work to be of different quality, then they will also experience different team processes.

Hybrid work is becoming increasingly widespread in the USA, where nearly fifty percent of remote-capable workers alternate between home and office. In the EU-27, regular and occasional hybrid workers account for approximately 25% of all employees (Parent-Thirion et al., 2025). This development, however, impacts not only on where or when employees will work, but also how they will work with their colleagues, and how the team will interact and coordinate its activities. Teams whose members alternate between in-site and remote work are being labelled “hybrid teams” and are defined as a combination of co-located and remote employees (Handke et al., 2025; Wiatr & Skowron-Mielnik, 2023). Differently from fully virtual teams, in which all components work remotely and from different locations, or traditional fully co-located teams, where all members work in the office, hybrid teams are composed of some employees that work remotely while the other colleagues work on-site, or in the office. Moreover, as remote work opportunities expand, patterns of office attendance are likely to fluctuate across days, with employees alternating between remote and on-site work. Thus, hybrid teams operate in an “in-between space”, where on-site staff and remote staff are continuously shifting or flipping (Handke et al., 2025). This fluidity sets hybrid teams as a qualitatively distinct form of team, with novel implications for organizations. Hybrid teams are also different from “partially distributed teams”, that is, teams composed of stable staff co-located and stable staff geographically dispersed (Huang & Ocker, 2006), and as such, they represent a different and more dynamic form of collaboration. Rather than being defined by stable patterns of presence, hybrid teams move back and forth between in-person and remote settings.

The alternation between working in the office and working remotely may create challenges for team functioning and management, such as coordination difficulties, communication gaps, or conflicts that have to be managed across multiple media (Mitchell & Brewer, 2021). In addition, hybrid teams, because of their mixed configuration, may be subjected to the formation of subgroups between in-office and remote members (Handke et al., 2025), the exclusion of remote workers from informal or strategic discussions, or slower planning and decision-making due to asynchronous communication (Buła et al., 2024; Mas & Pallais, 2020). Consequently, effective hybrid team management requires careful planning and intentional design of the in-person, remote, and hybrid interactions (Buła et al., 2024; Donati et al., 2025)

Consistent with the Team Perceived Virtuality (TPV) model (Handke et al., 2021), hybrid teamwork is shaped not only by objective features such as geographic dispersion, insite or remote work schedules, or technology use, but also by team members’ collectively constructed experiences of virtuality. This model conceptualizes team perceived virtu-ality as a shared affective–cognitive emergent state characterized by collectively experienced distance and perceived information deficits.

Collectively experienced distance refers to shared feelings of emotional inaccessibility or unavailability, while collectively experienced information deficits refer to shared perceptions of inadequate information exchange and difficulties in converging on meaning. The construct of TPV captures a deficit-oriented state with higher levels of perceived distance and information deficits related to more negative and lower levels of team functioning (Handke et al., 2021). Considering higher and lower values of perceived distance and information deficits, Handke et al., (2021) describe four different TPV states, going from a less to a more effective team functioning. Using the same approach, and relying on the QoT model, we argue that the individual perception of the quality of one’s own remote work may be related to the individual perception of the functioning of the hybrid team. Specifically, we argue that hybrid work arrangements, characterized by good quality in terms of technologically well-equipped offices, adequate remote workstation, a good flexibility in the management of one’s working time, and managerial practices that provide role clarity and transformational leadership, will be related to positive teamwork processes. The teamwork processes we consider in this study are team identification, perceptions of collective self-efficacy, know-ledge sharing, team efficiency, and satisfaction with the hybrid team.

In particular, considering the four core domains of QoT (Miglioretti et al., 2021, 2022) it is reasonable to expect that offices adequately equipped for remote work might provide good quality and a variety of work settings, such as collaborative and informal spaces that enhance and promote sociability, and facilitate task information and knowledge exchange among team members. Employees working in such physical conditions may also experience higher team identification, sense of belonging to the team and satisfaction with the hybrid team. Functional remote workstations may be effective not only for the individual employee but may also represent an enabling resource for team functioning: by reducing environmental distractions, adequate workspaces allow employees to engage more reliably in interactions with his/her team and to contribute their expertise without friction. Over time, repeated experiences of smooth coordination and successful task execution even when working remotely foster beliefs in the team’s collective capability and enhance perceptions of team efficiency across in-office and remote settings. Flex-time represents a temporal autonomy resource that may shape how individual effort is coordinated within hybrid teams. By allowing employees to align their work schedules with personal and task-related demands, flex-time is related to individual job satisfaction (Boccoli et al., 2022), and we argue it might also support perceptions of being an effective team. At the same time, team efficiency depends on the presence of shared norms that balance individual flexibility with coordination needs. Finally, an engaging management, that establishes clear activities and objectives combined with transformational leadership, may improve employees’ comprehension of individual and team goals, clarify the role of each member and the contribution s/he can give to the goals, and encourage reciprocal support, thus promoting not only knowledge sharing but also a sense of collective self-efficacy. Transformational leadership considered a crucial resource for teleworking contexts (Miglioretti et al., 2022), may support motivation and shared commitment, thereby facilitating knowledge sharing and identification with the hybrid team.

However, if the leader is not effective in maintaining cohesiveness, or team members make individual choices using their autonomy to select work locations that maximize their resource gains (such as, for instance, their own work–life balance, ergonomic conditions, or role clarity) and minimize resource losses (e.g., commuting time, workplace stress), uneven patterns of co-location across team members may increase the likelihood of subgroup formation. Having subgroups of hybrid workers, or individual hybrid workers, may also reduce the visibility of team members’ efforts, restrict opportunities for knowledge sharing, or weaken team identification and collective self-efficacy (Hopkins & Bardoel, 2023). If knowledge about the adequate use of communication technologies is not shared, coordination and cohesion decrease because of the misalignment of expectations about interaction channels and timing (Begemann et al., 2024). Finally, if technology or remote work environments are inadequate, barriers to communication and knowledge sharing across workers located in different workplaces are hindered. Ineffective collaboration tools and inadequate facilities may increase individual workers’ perception of emotional distance and informational deficits of the team, increasing perception of collective inefficacy, team inefficiency and low satisfaction for the functioning of the hybrid team.

Considering the core resources of the QoT model, it is reasonable to expect that employees working in a context that provide an effective physical environment, a functional remote workstations, together with autonomy in managing working schedules and support from leaders who define clear roles and motivating work goals, will experience hybrid teamwork as more satisfying and more adequate for exchanging information, performing tasks, and overcoming work difficulties. Accordingly, we hypothesize that different profiles of hybrid work quality are associated with distinct perceptions of hybrid team processes-specifically team identification, perceived team knowledge sharing, and beliefs about collective team self-efficacy-as well as with team outcomes, namely satisfaction with hybrid teamwork and perceived team effectiveness.

Accordingly, the following hypothesis is proposed:

**Hypothesis 2.** *Hybrid work profiles characterised by higher perceived levels across the four core quality resources are associated with more favourable perceptions of hybrid team processes and team outcomes, compared to profiles characterised by perceived lower levels of hybrid work quality*.

## Method

### Procedure

Data were collected through a web-based survey administered via Qualtrics between April and October 2025. Participants were employees from four medium-to-large organizations, including three private companies (automotive, consulting, and payroll/workforce services) and one public administration. All participants were involved in hybrid work as part of their standard job arrangements and occupied a wide range of professional roles and positions across different departments. The survey link was distributed through an organizational email sent by the Human Resources department of the participating organizations. Access to the data was restricted to the research team involved in the project. Completion of the questionnaire required approximately 10– 15 minutes. The study protocol was reviewed and approved by the Committee for Research Evaluation [Anonymized]. All participants provided informed consent, and data were handled in accordance with current ethical and data protection regulations.

### Participants

The final sample consisted of 747 employees. Because of missing responses on one or more study variables, 719 participants were retained for the analyses (45.1% men, 54.1% women, 0.8% other), with a mean age of 43.7 years (SD = 10.7; range = 22–65). Average organizational tenure was 16.1 years (SD = 10.7; range = 0.8–43). With respect to education, 37.2% of participants reported primary or secondary education, whereas 62.4% held an academic or postgraduate degree. Regarding organizational role, 22.2% of respondents occupied managerial positions, while 77.2% were non-managerial employees. In terms of remote work intensity, 33.6% of participants reported working remotely fewer than four days in the previous 15 days, 43.1% reported between four and six remote working days, and 23.3% reported seven or more remote working days during the same period. The Margheritti et al.: Hybrid Work Profiles and Team Functioning number of remote working days was categorized into three categories (less than four days, between four and six days, equal or more than 7 days in two weeks). This classification is based on remote work data reported in previous survey conducted at the national level in Italy (Assolombarda, 2024; Confindustria, 2024; Osservatori Digital Innovation del Politecnico di Milano, 2024)

### Measures

#### Quality of Telework measures

The quality of telework was measured with 38 items assessing the four core components of QoT model through seven factors (Miglioretti et al., 2022):

(1) *Quality of offices in the organisation*, consisting of two dimensions: Office quality measured by six items (α = .87) and Office flexibility composed of four items (α = .68). Example items are ‘My space within my organisations is ergonomic’, and ‘During my working day in the company, I can use informal spaces to have a chat’ for office quality and office flexibility, respectively. The response scale ranges from 1 strongly disagree to 5 strongly agree.

(2) *Functional remote workstations*, consisting of two dimensions: Suitability of remote workstations measured by six items (α = .91), and Healthiness of remote workstations composed of four items (α = .88). Example items are ‘The seating positions in the place where I work outside my organisations are adequate (e.g. comfortable chairs, good postural support)’, and ‘The place outside the central office where I work is free of excessive noise’, respectively, for the two dimensions. The response scale ranges from 1 strongly disagree to 5 strongly agree.

(3) *Flextime*, operationalised with one dimension of six items (α = .83). An example item is ‘I manage the planning of my day independently’. The response scale ranges from 1 never to 5 always.

(4) *Enabling management*, consisting of two dimensions: Clarity of objectives, measured by four items (α = .92), and Transformational leadership, measured with eight items (α = .97). Example items are ‘I am clear about my job tasks and my responsibilities’, and ‘My supervisor provides guidance when I need it’, respectively, for the two dimensions. The response scale ranges from 1 never to 5 always.

#### Hybrid team measures

The following validated scales were used to assess hybrid team processes and outcomes.

(5) *Team identification* was measured using four items from Frenzel et al. (2023) (e.g., “I feel that I am part of this team/office”; “I feel that I have meaningful relationships within this team/office”) (α = .93).

(6) *Team knowledge sharing* was assessed with three items from Bock et al. (2005) (e.g., “At work, I share my experience or know-how with my colleagues”) (α = .93).

(7) *Team collective self-efficacy* was assessed with two items from Frenzel et al. (2023) (e.g., “In this team, we are able to overcome difficulties together”) (α = .93).

(8) *Team efficiency* was measured using five items from Hoegl and Gemuenden (2001) (e.g., “Task completion did not take more time than initially planned”; “The outcomes achieved by the team/office do not require further improvement”) (α = .83).

(9) *Satisfaction with hybrid teamwork* was measured using four items adapted from Aguilera, Bastarrica, and Simmonds (2017) (e.g., “This way of working allows me to easily contact my team/office colleagues”; “I feel comfortable working in this mode”) (α = .86).

### Data Analysis

The analyses performed to address our research question consisted of two steps: first a latent profile analysis was run to observe the different profiles of quality of hybrid work; then, the association between profiles and team variables was tested. The first step was performed in Mplus (version 8), and the second step was performed using SPSS (version 29).

*Latent Profile Analysis*. To identify the groups that best describe the heterogeneity within the current sample with respect to quality of hybrid work, we performed a latent profile analysis (LPA), including the resources of the QOT Model as observed indicators. We examined fit indices of measurement models, beginning with one class and adding classes incrementally. We stopped estimating additional classes when empirical under-identification or convergence issues were encountered. As suggested by Sorgente et al. (2019), selecting the optimal fitting model(s) was based on absolute and relative fit indices.

Absolute model fit was evaluated using the likelihood ratio chi-square test (LRT2) and standardized residuals. For the LRT2, a non-significant p-value (*p* > .05) indicates adequate model fit; therefore, the preferred model is the most parsimonious one that does not reject the null hypothesis. Standardized residuals were evaluated across all observed– expected cell comparisons; model fit was considered acceptable when fewer than 5% of the total standardized residuals exceeded |3| (Masyn, 2013).

Relative model fit was assessed using both descriptive and inferential criteria. Descriptive indices included AIC, CAIC, AWE, BIC, and SABIC, with lower values indicating better fit. Based on BIC, the approximate Bayes factor (BF) and the correct model probability (cmP; Nagin, 1999) were also examined. Models with BF > 3 and higher cmP values were preferred, with cmP > .10 indicating plausible candidate models.

Inferential comparisons between *k*-1 and *k* class models were conducted using the VLMR-LRT, adjusted LMR-LRT, and BLRT. A significant *p*-value indicates that the *k*-class model provides a better fit than the model with one fewer class; otherwise, the more parsimonious solution was retained.

After model selection, classification quality was evaluated using entropy, class proportions (πk), modal class assignment proportions (mcaPk), average posterior probabilities (avePPk), and odds of correct classification (OCCk). Classification was considered adequate when entropy approached 1, mcaPk fell within the 95% CI of πk, avePPk ≥ .70, and OCCk > 5 (Masyn, 2013; Sorgente et al., 2024)).

*Relationship between profiles and Hybrid Team variables*. After identifying the best LPA model, we saved the most likely class membership for each individual having an observed variable representing each participant’s membership in a specific quality of hybrid work group. Following the classify-analyze approach (Ulbricht et al., 2018), we used this class membership variable to test the relationship quality of hybrid work has with respondents’ perception of the functioning of hybrid work arrangements.

In particular, we conducted a series of one-way analyses of variance (ANOVAs) to examine whether membership in different hybrid work quality classes was associated with our outcomes. Post hoc comparisons were performed using the Bonferroni correction.

## Results

Table 1 reports the descriptive statistics for the Quality of Telework (QoT) variables included in the latent profile analysis.

**Table 1:** Descriptive statistics QoT-q variables

Variable	Min	Max	M	SD
Office Flexibility	1.00	5.00	3.51	0.78
Office Quality	1.00	5.00	3.87	0.75
Remote Work Healthiness	1.00	5.00	4.36	0.63
Flextime	1.00	5.00	3.39	0.92
Clarity of Objectives	1.00	5.00	4.28	0.73
Transformational Leadership	1.00	5.00	3.99	0.92

**Note.** *M* = mean; *SD* = standard deviation.

### Identification of Latent Profiles

We estimated a series of latent profile models ranging from one- to eight-profile solutions. Models with more than four profiles were not retained because the best loglikelihood was not replicated. In addition, solutions with five or more profiles were discarded because the smallest profile included less than 1% of participants, which is commonly considered an unacceptably small class size for substantive evaluation and reliable estimation (Nylund-Gibson & Masyn, 2016). As shown in Table 2, the four-profile solution provided the most satisfactory balance between model fit and parsimony. Specifically, this solution was characterized by lower AIC, BIC, CAIC, and AWE values compared to models with fewer profiles, as well as by adequate model stability.

**Table 2:** Absolute and Relative Model Fit Indices for Latent Class Models

Model	LL	AIC	CAIC	AWE	BIC	SSBIC	SIC	BF	cmP	VLMR-LRT test	LMR-LRT test	BLRT test
1-class	-5.696,739	11421,478	11499,56806	11619,65812	11485,56806	11441,11419	-5742,78403	0,00	0			
2-class	-5.450,494	10944,988	11067,70095	11256,4139	11045,70095	10975,84488	-5522,850475	0,00	0,00	**P=.03**	**P= .03**	**P= .001**
3-class	-5.352,319	10764,638	10931,97384	11189,30968	10901,97384	10806,71556	-5450,98692	0,00	0	P=0.14	P=0.14	**P= <.001**
4-class	-4.826,244	9728,488	9940,446732	10266,40546	9902,446732	9781,786242	-4951,223366	0,00	0	P=0.14	P=0.15	**P= <.001**
5-class	-4.039,508	8171,016	8427,597622	8822,179245	8381,597622	8235,534924	-4190,798811	0,00	0,00	P=0.23	P=0.24	**P= <.001**
6-class	-3.990,453	8088,906	8390,110513	**8853,315027**	8336,110513	8164,645607	-4168,055257	0,00	0,00	P=0.43	P=0.43	**P=0.000**
7-class	-3.934,733	7993,466	7931,466	8871,120808	8277,293404	8080,426289	-4138,646702	0,16	0,02	p=0.47	p=0.47	**P=0.000**
8-class	**-3.906,568**	**7953,136**	**7883,136**	8944,03659	**8273,586295**	**8051,316971**	-4136,793148	0,00	**0,98**	P=0.35	P=0.35	**P=0.000**

LL = loglikelihood; AIC = Akaike information criterion; CAIC = Consistent AIC; AWE = Approximate Weight of Evidence criterion; BIC = Bayesian information criterion; SSBIC = sample-size adjusted BIC; BF = Bayesian factor; cmP = approximate correct model probability; VLMR-LRT = Vuong-Lo-Mendell-Rubin likelihood ratio test; LMR-LRT = Lo– Mendell–Rubin likelihood ratio test; BLRT = bootstrapped likelihood ratio test. Fit indices corresponding to the best-fitting model(s) are shown in bold.

Classification diagnostics further supported the adequacy of the four-profile solution. As reported in Table 3, the entropy value was high (E = .93), indicating good classification accuracy, and average posterior probabilities suggested that individuals were clearly assigned to their respective profiles. Overall, the four-profile solution identified well-differentiated and theoretically meaningful profiles of hybrid work quality and was therefore retained for further analyses.

**Table 3:** Classification Diagnostics for the Three-Class and Four-Class Model

Entropy (E)	Class (N)	CP	mcaP	AvePP	OCC
0.792	Class 1 (46)	.064 (.046 .081)	0.064	0.999	146235.38
	Class 2 (298)	.414 (.378 .449)	0.414	0.999	14153.17
	Class 3 (92)	.137 (.066 .207)	0.127	0.881	46.64
	Class 4 (283)	.384 (.307 .460)	0.393	0.937	23.86

*Note*. E = Entropy; CP = class proportion; mcaP = modal class assignment proportion; avePP = average posterior probability; OCC = odds of correct classification.

**Figure 1:**
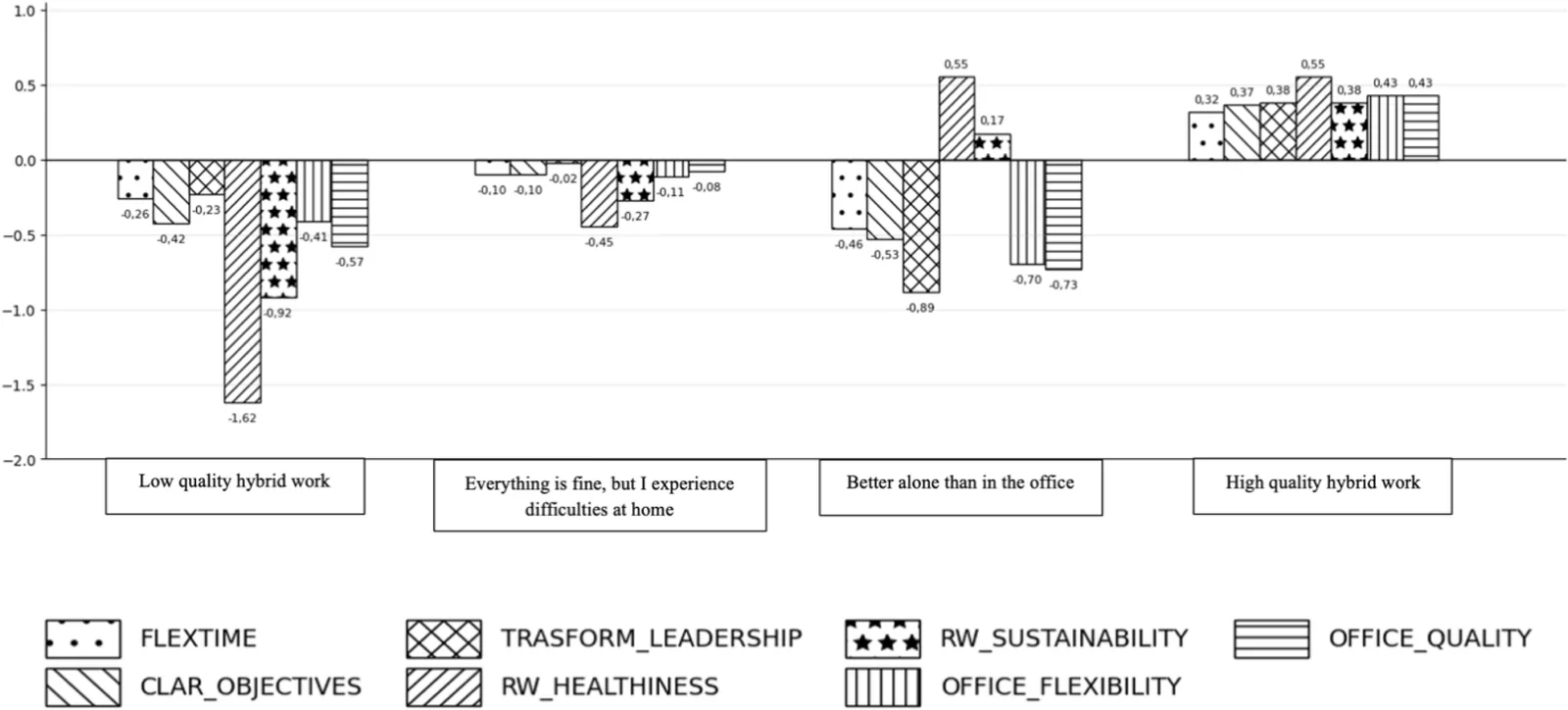
Representation of the four hybrid work quality profiles. *Note*. Values reported in the figure are standardised means.

The four latent profiles (see Figure 1) differed in terms of perceived hybrid work quality and were distributed, and labelled, as follows: *High-quality hybrid work* (n = 283; 39.4%), *Everything is fine, but I experience difficulties at home* (n = 298; 41.4%), *Better alone than at the office* (n = 92; 12.8%), and *Low-quality hybrid work* (n = 46; 6.4%). Descriptive statistics are reported in Table 3.

The *High-quality hybrid work* profile (displayed on the right-hand side of the Figure 1) was characterized by high levels across all dimensions of hybrid work quality, reflecting a consistently positive agile work experience. *The Everything is fine, but I experience difficulties at home* profile showed generally adequate levels of hybrid work quality combined with lower quality in the home context. *The Better alone than at the office* profile was characterized by an unbalanced pattern, with relatively positive evaluations of individual work-related aspects but lower scores on dimensions related to organizational and relational resources. Finally, the *Low-quality hybrid work* profile (displayed on the left-hand side of the Figure 1) reported consistently low levels across all hybrid work quality dimensions, indicating an overall unfavorable hybrid work experience.

### Relationship between the quality of hybrid work profiles and hybrid team variables

The hybrid work profile membership was used as the independent variable in a series of one-way ANOVAs examining differences in team processes and outcomes across the four profiles. Descriptive statistics by latent profile are reported in Table 4.

**Table 4:** Descriptive statistics by latent profile on hybrid team

Profile	Variable	N	Min	Max	M	SD
**Low-quality hybrid work**	Team Identification	45	1.00	5.00	3.64	1.04
	Team Knowledge Sharing	42	2.00	7.00	5.60	1.17
	Team Collective Efficacy	45	1.00	5.00	3.61	1.04
	Team Hybrid Mode	45	1.00	5.00	3.64	0.81
	Team Efficiency Efficacy	41	1.40	5.00	3.45	0.72

**Everything is fine, but I experience difficulties at home**	Team Identification	290	1.50	5.00	4.00	0.74
	Team Knowledge Sharing	286	1.00	7.00	5.97	1.28
	Team Collective Efficacy	290	1.00	5.00	4.03	0.75
	Team Hybrid Mode	290	1.50	5.00	4.14	0.65
	Team Efficiency Efficacy	276	2.20	5.00	3.61	0.56

**Better alone than at the office**	Team Identification	90	1.00	5.00	3.51	1.04
	Team Knowledge Sharing	89	1.00	7.00	5.93	1.12
	Team Collective Efficacy	90	1.00	5.00	3.61	1.04
	Team Hybrid Mode	89	2.25	5.00	4.38	0.62
	Team Efficiency Efficacy	85	1.00	5.00	3.48	0.80

**High-quality hybrid work**	Team Identification	275	2.00	5.00	4.31	0.65
	Team Knowledge Sharing	274	1.00	7.00	6.41	0.70
	Team Collective Efficacy	275	2.00	5.00	4.36	0.67
	Team Hybrid Mode	275	2.75	5.00	4.55	0.57
	Team Efficiency Efficacy	272	1.20	5.00	3.89	0.62

The results revealed statistically significant differences across the four hybrid work quality profiles on all team-related outcomes. Specifically, significant omnibus effects were observed for team identification (*F*(3, 743) = 29.65, p < .001, η^2^ = .11, ω^2^ = .11), team knowledge sharing (F(3, 743) = 13.03, p < .001, η^2^ = .05, ω^2^ = .05), satisfaction with hybrid teamwork (*F*(3, 743) = 37.45, p < .001, η^2^ = .14, ω^2^ = .13), team collective self-efficacy (*F*(3, 743) = 28.69, p < .001, η^2^ = .11, ω^2^ = .11) and team efficiency (*F*(3, 743) = 15.59, p < .001, η^2^ = .06, ω^2^ = .06). Overall, effect sizes ranged from moderate to large, indicating meaningful differences in perceived team functioning across hybrid work profiles.

Post-hoc comparisons with Bonferroni adjustment revealed a consistent and theoretically interpretable pattern. Employees belonging to the *High-quality hybrid work* profile reported the highest levels across all team-related variables. In contrast, the *Low-quality hybrid work* profile showed significantly lower scores, particularly in team knowledge sharing, satisfaction with hybrid teamwork, and team efficiency. Compared to the *High-quality hybrid work* profile, employees in the *Low-quality hybrid work* profile reported lower team identification (ΔM = −0.670, *p* < .001), lower team knowledge sharing (ΔM = −0.803, *p* < .001), lower satisfaction with hybrid teamwork (ΔM = −0.902, *p* < .001), lower team collective self-efficacy (ΔM = −0.753, *p* < .001), and lower team efficiency (ΔM = −0.439, *p* < .001).

The *Everything is fine, but I experience difficulties at home* profile displayed intermediate levels of team functioning across most dimensions. Compared with the *High-quality hybrid work* profile, this group reported significantly lower scores in team identification (ΔM = −0.309, *p* < .001), collaborative efficacy (ΔM = −0.336, p < .001), and satisfaction with the hybrid mode of teamwork (ΔM = −0.408, *p* < .001). In contrast, relative to the *Low-quality hybrid work* profile, participants in this group reported significantly higher levels of team identification (ΔM = 0.494, *p* < .001), collaborative efficacy (ΔM = 0.422, *p* < .001), knowledge sharing (ΔM = 0.439, *p* < .001), and efficiency-related efficacy (ΔM = 0.280, *p* < .001). No significant differences emerged between these two profiles with respect to task interdependence.

Finally, the *Better alone than at the office* profile exhibited a distinct pattern across the examined dimensions of team functioning. Compared with the *High-quality hybrid work* profile, employees in this group reported significantly lower levels of team identification (ΔM = −0.804, *p* < .001), collaborative efficacy (ΔM = −0.758, p < .001), knowledge sharing (ΔM = −0.481, *p* = .001), and efficiency-related efficacy (ΔM = −0.408, *p* < .001), while also reporting lower satisfaction with the hybrid mode of teamwork (ΔM = −0.170, ns). Relative to the *Everything is fine, but I experience difficulties at home* profile, this group showed significantly lower scores in team identification (ΔM = −0.494, *p* < .001) and collaborative efficacy (ΔM = −0.422, *p* < .001), whereas no significant differences emerged with respect to task interdependence. In contrast, no significant differences were observed between this profile and the *Low-quality hybrid work* profile across most dimensions, except for satisfaction with the hybrid mode of teamwork, which remained significantly higher in the Better alone than at the office profile (ΔM = 0.170, p = .159).

## Discussion

Drawing on a person-centred perspective, this study first contributes to the hybrid work literature by identifying distinct profiles of hybrid workers based on the Quality of Telework (QoT) model (Margheritti et al., 2023; Miglioretti et al., 2022). Moreover, it sheds light on how these profiles are associated with employees’ perceptions of team functioning in hybrid work contexts. From a methodological point of view, this study contributes to person-oriented research by demonstrating the value of examining perceived configurations of work-related resources rather than isolated predictors. The findings highlight that hybrid work experiences are not uniformly distributed across individuals but instead form distinct profiles associated with different patterns of team functioning.

Regarding Hypothesis 1 (H1), we identified four hybrid worker profiles based on four core resources, confirming the configurations previously identified by Miglioretti et al. (2023) during the peak of the COVID-19 pandemic. Overall, the identified profiles highlight that the quality of hybrid work does not depend on the mere availability of flexibility, but rather on a balanced configuration of organizational, managerial, and infrastructural resources.

The “*Low-quality hybrid work*” profile, albeit numerically small, comprises workers who perceive the absence of these resources across all the QoT Model domains (i.e., agile offices within organisations, functional remote workstations, flex-time, and enabling management). According to the literature, this profile severely undermines the hybrid work experience, suggesting that partial or poorly implemented hybrid arrangements may be particularly detrimental at both the individual and organizational level (Miglioretti et al., 2022).

The two intermediate profiles offer a more nuanced picture. The “*Everything is fine, but I experience difficulties at home*” profile, which represents the largest share of workers, points to a critical vulnerability of hybrid work models: even when offices within organisations, leadership, and flexibility are perceived positively, inadequate remote infrastructures and difficulties in managing domestic demands can substantially limit the perceived quality of hybrid work (Nakrošienė et al., 2019). This finding suggests that hybrid work may unintentionally reproduce or exacerbate inequalities linked to living conditions and private resources (Toscano et al., 2025). Importantly, this profile indicates that the effectiveness of hybrid work cannot be fully ensured through organizational and managerial resources alone when home-based conditions remain structurally unequal. Indeed, recent evidence on hybrid workers indicates that perceptions of home office ergonomics and comfort, relative to traditional office settings, shape both employees’ engagement in hybrid work and their online performance (Donati et al., 2025).

Conversely, the “*Better alone than in the office*” profile reveals an opposite but equally problematic configuration. Although employees in this profile report adequate remote workstations, the absence of agile office environments, flextime, and enabling leadership undermines the conditions that the literature identifies as essential for high-quality hybrid work. Research consistently shows that agile offices are not merely physical locations, but socio-technical systems that must be equipped with appropriate infrastructures and technologies, ensure ergonomic and healthy working conditions, and support collaboration within hybrid teams composed of both remote and on-site workers. When offices are not designed for these purposes, they fail to facilitate coordination, social exchange, and collective work processes, thereby reducing the integrative potential of hybrid work. Similarly, the lack of flex-time deprives employees of autonomy over the temporal organization of their work. Further evidence suggests that the advantages of hybrid working arrangements are contingent on the degree of schedule autonomy embedded in employees’ jobs (Allen et al., 2015). Finally, without enabling leadership, characterized by clear objectives, delegation, and transformational behaviours that provide direction, shared goals, and motivational support (Bass, 1990; De Leede & Kraijenbrink, 2014; Peters et al., 2014), hybrid work risks becoming a fragmented and individualised practice rather than an effective organizational model.

Finally, the “*High-quality hybrid work*” profile comprises workers who perceive the presence of these resources across all the QoT Model domains (i.e., agile offices within organisations, functional remote workstations, flex-time, and enabling management), showing that a positive hybrid work experience emerges only when resources are available simultaneously at the individual and organizational levels. Taken together, these four profiles suggest that hybrid work quality is a systemic phenomenon: deficiencies in any single domain, whether domestic, organizational, or managerial, can compromise the overall effectiveness and sustainability of hybrid work arrangements.

Regarding Hypothesis 2 (H2), the present findings provide robust empirical evidence that workers’ experiences of team functioning significantly differ according to their latent profile membership, revealing clearly differentiated perceptions of teamwork processes and outcomes. Overall, the results highlight the existence of distinct patterns in employees’ reported experiences of hybrid teamwork, emphasizing the relevance of adopting a person-centered analytical perspective.

Across all team-related dimensions, the *High-quality hybrid work* profile consistently displayed the most positive pattern of perceptions, characterized by elevated levels of team identification, knowledge sharing, collective team efficacy, perceived team efficiency, and satisfaction with hybrid work arrangements. Workers belonging to this profile perceive their team as functional, cohesive, and highly effective, suggesting that favorable agile work conditions are associated not only individual well-being but also positive team dynamics and outcomes.

Conversely, the *Low-quality hybrid work* profile systematically exhibited the most critical pattern, reporting the lowest scores across all variables. This configuration reflects perceptions of limited social integration within the teammanifested in lower team identification and collective self-efficacy-together with reduced knowledge sharing and diminished evaluations of team effectiveness and satisfaction with hybrid teamwork. These findings suggest that poor agile work quality is associated with dysfunctional team experiences, potentially undermining both relational and performance-related processes.

From a theoretical point of view, the adoption of the Team Virtuality Perception (TVP) model (Handke et al., 2021) further enabled a more nuanced interpretation of these two profiles findings, illustrating how distinct QoT configurations correspond to varying degrees of perceived team virtuality. High-quality agile work conditions appear conducive to perceptions of stronger interpersonal closeness, more effective information sharing, and reduced team virtuality. In contrast, workers in the low-quality profile reported higher perceived team virtuality, weaker affective bonds, and diminished knowledge exchange. This pattern suggests that hybrid work design is related not only to individual work experiences but also to how employees perceive the structure and functioning of team interactions.

Going to the findings about the other two intermediate profiles, the “Everything is fine, but I experience difficulties at home” and “Better alone than at the office” profiles displayed differentiated and theoretically meaningful patterns. The former profile showed moderately positive levels of team identification, collective self-efficacy, knowledge sharing, perceived team effectiveness, and satisfaction. This configuration appears to reflect generally favorable agile work conditions, particularly regarding organizational agile offices, flex-time arrangements, and enabling management practices, coupled with perceived difficulties in the homebased work environment. In contrast, the “*Better alone than at the office*” profile was characterized by lower team identification and collective self-efficacy, yet relatively positive perceptions of hybrid work satisfaction. This pattern seems to emerge from less favorable organizational conditionssuch as leadership quality, flexibility, goal clarity, and office physical environment-combined with positive evaluations of functional remote workstations. Together, these conditions may foster a preference for remote individual work, reducing workers’ sense of belonging to a collective team and increasing their inclination to operate autonomously. These findings invite further theoretical refinement by integrating hybrid work design more explicitly into models of team functioning. In particular, the apparent contrast between the two intermediate profiles highlights the necessity of balancing managerial, organizational, and technological dimensions of agile work. Effective hybrid work design should not focus solely on leadership practices, autonomy, and organizational support but also ensure adequate material and ergonomic resources in remote work environments. At the same time, hybrid contexts characterized by limited managerial support and autonomy, despite offering high-quality remote resources, may inadvertently foster individualistic and potentially disengaged work behaviors, as suggested by the “*Better alone than at the office*” profile.

Furthermore, these results resonate with the Conservation of Resources (COR) theory, which posits that individuals are motivated to preserve, acquire, and protect valuable resources (Hobfoll, 1989; Hobfoll et al., 2018). In hybrid work contexts, employees may strategically select work locations that maximize personal resource gains-such as work–life balance, ergonomic comfort, and role clarity-while minimizing resource losses, including commuting time and workplace stress (Donati et al., 2025). Although adaptive at the individual level, such strategies may produce uneven patterns of co-location, increasing the likelihood of subgroup formation and reducing opportunities for team-wide interaction (Handke et al., 2025). Over time, this dynamic may weaken team identification, collective efficacy, and reciprocal knowledge sharing, ultimately shaping team processes and outcomes. At the team level, persistent spatial fragmentation between hybrid workers may foster localized subgroups, reinforcing in-group dynamics at the expense of broader team cohesion and integration (Handke et al., 2025). These processes may be linked to exert long-term effects on relational quality, information exchange, and consequently on their perceptions of hybrid team functioning and effectiveness.

Overall, from a theoretical standpoint, the results discussed so far align with person-centered approaches in organizational research, which stress the importance of examining naturally occurring constellations of perceived work experiences rather than isolated predictors (Morin et al., 2018). The pronounced differences in hybrid work satisfaction underscore the central role of team processes in shaping employees’ overall experiences of flexible work arrangements. When hybrid work is perceived as high-quality, workers tend to report more positive perceptions of team functioning, whereas deteriorating hybrid work conditions are associated with less favorable teamwork perceptions. Specifically, positive evaluations of enabling managementsuch as transformational leadership and goal clarity-together with temporal flexibility, appear to support stronger team identification, enhanced knowledge sharing, greater collective efficacy, and higher perceived effectiveness. Conversely, differentiated experiences concerning office and remote work environments generate distinct patterns of team perception, as observed in the two intermediate profiles.

Finally, the present findings contribute to the advancement of the Quality of Telework (QoT) model by showing that hybrid work quality is systematically related to perceptions of hybrid teamwork processes and outcomes. The clear differentiation between high- and low-quality hybrid work profiles underscores the pivotal role of work design in shaping collective experiences. Moreover, the emergence of specific configurations-such as “Better alone than at the office” and “Everything is fine, but I experience difficulties at home”-suggests that distinct combinations of QoT dimensions are associated with qualitatively different perceptions of teamwork. Whereas the former group appears primarily satisfied due to positive evaluations of the remote workstation, despite less favorable organizational conditions, the latter profile is characterized by strong organizational support coupled with difficulties in the home-based work environment. These findings indicate that the various dimensions of QoT show differentiated and, at times, opposing associations with teamwork perceptions.

### Practical implications

These findings highlight the importance of intentionally designing high-quality hybrid work environments that support team functioning. Rather than focusing solely on the presence or intensity of hybrid work, organizations should consider how different configurations of perceived resources shape employees’ experiences and their perceptions of teamwork.

The profiling approach developed in this study offers a useful diagnostic tool for identifying heterogeneous hybrid work experiences within organizations. Understanding profile membership can help organizations tailor interventions aimed at improving both the quality of hybrid work and team-related outcomes.

At the individual level, differentiated actions may be implemented depending on workers’ profile membership. For example, ergonomic interventions and investments in improving the quality of remote workstations appear particularly relevant for employees belonging to the “*Everything is fine, but I experience difficulties at home*” profile. Conversely, for workers in the “*Better alone than at the office*” profile, efforts should focus primarily on strengthening enabling managerial practices, such as clearer goal-setting, supportive leadership, and increased opportunities for participation and autonomy. Such targeted actions can mitigate profile-specific challenges and promote more balanced and sustainable hybrid work experiences.

At the group level, team-building initiatives, structured opportunities for communication, shared goal setting, and enhanced task coordination may be especially beneficial in teams characterized by a high prevalence of “*Low-quality hybrid work*” or “*Better alone than at the office*” profiles. These interventions may help reinforce interpersonal connections, facilitate knowledge sharing, and foster a stronger sense of collective identity, thereby improving team functioning and performance.

Furthermore, the results underscore the importance of actively managing team composition. Managers who are aware of the distribution of profiles within their teams can more effectively anticipate potential collaboration challenges and adapt leadership strategies accordingly. For instance, granting greater autonomy and flexibility in work methods may be particularly beneficial for members of the “*Better alone than at the office*” profiles, whereas improving access to functional and ergonomic remote workstations may better support employees in the “*Everything is fine, but I experience difficulties at home*” profile. Again, for the “*Lowquality hybrid work*” profile, the results suggest that a more systemic and multi-target approach could be beneficial. This may include initiatives to improve the ergonomic and qualitative aspects of both on-site and remote work environments, along with enhancements to managerial practices that better support hybrid workers. In particular, clearer goal-setting, more supportive leadership behaviours, and organizational practices that facilitate autonomous time management may help create more effective and sustainable hybrid work conditions. Attending to how different profiles interact within the same team and encouraging mutual support and adaptive behaviors can enhance team effectiveness even when structural levels of virtuality remain unchanged.

Finally, these findings highlight the importance of fostering ongoing reflection among managers and team members regarding their perceptions of team virtuality. Rather than assuming static or predetermined effects of hybrid work arrangements, teams should regularly discuss the factors shaping their current levels of perceived virtuality and collaboratively identify strategies to maintain or modify these dynamics in ways that enhance effectiveness. In this regard, participatory approaches such as the SMART model method (Parker & Knight, 2024) offer a promising framework for collaboratively redesigning hybrid work practices, placing employees’ perspectives at the center of organizational decision-making, and promoting more sustainable, flexible, and person-focused workplaces.

### Limitations and Future Research Directions

Despite the innovative contribution, the present study has some limitations. First, the person-centered approach represents a key strength of the present study, as it allows for the identification and characterization of individuals based on their subjective perceptions of hybrid work. At the same time, such perceptions are inevitably embedded within specific organizational and remote-work contexts. Consequently, the structure, prevalence, and interpretation of the identified profiles may vary across organizational, occupational, or cultural settings. Consistent with prior person-centered research in other domains and sectors (e.g., the financial sector; (Miglioretti et al., 2022), our findings suggest that while similar profile configurations may emerge across studies, their distribution and functional meaning are likely to be contextdependent. In this regard, latent profile analysis offers valuable insights into latent heterogeneity, while also calling for systematic replication across diverse samples to strengthen the robustness and generalizability of profile solutions. From this perspective, differences observed in teamwork perceptions across profiles should be interpreted as contextually grounded patterns rather than universally generalizable effects. The causal interpretations -particularly regarding the associations between Quality of hybrid work dimensions and team-related processes and outcomes - should be approached with appropriate caution. Future research should therefore aim to replicate and extend these findings across a wider range of organizational and economic sectors, characterized by greater heterogeneity in agile work practices and hybrid work arrangements. Such efforts would contribute to a more comprehensive assessment of the stability, transferability, and theoretical relevance of the identified profiles and their associated patterns of team functioning.

Second, the cross-sectional design of the present study limits inferences about the temporal stability and developmental dynamics of the identified profiles. Longitudinal research is therefore warranted to examine whether profile membership reflects relatively stable configurations or dynamic states that evolve in response to changing organizational, technological, and relational conditions, as well as to explore potential transitions between profiles over time. In addition, experimental and intervention-based studies could assess whether changes in perceived agile work quality-such as modifications in leadership practices, work flexibility, office design, or remote-work resources-lead to shifts in profile membership and, in turn, to changes in hybrid team processes and outcomes. Finally, integrating Quality of hybrid work dimensions within longitudinal and process-oriented frameworks would offer deeper insights into the mechanisms underlying profile formation and transformation, thereby advancing theoretical models of hybrid work by capturing the dynamic and reciprocal relationships between work design, individual experiences, and team dynamics.

Finally, all measures relied exclusively on self-reported data, which may introduce common method variance, social desirability bias, and perceptual distortions. Although selfreports are particularly suitable for capturing subjective experiences and perceptions, future research would benefit from incorporating objective and organizational archival data. For example, objective indicators of remote and office workstation ergonomics and environmental sustainability, formal records of goal-setting procedures, indicators of team performance (e.g., goal attainment and adherence to deadlines), and digital traces of communication frequency, distribution, and knowledge-sharing patterns among team members could provide complementary evidence. The integration of multi-source and multi-method data would allow for a more comprehensive, robust, and ecologically valid assessment of both hybrid work quality and hybrid team functioning.

## Conclusion

Drawing on a person-oriented perspective, this study advances understanding of hybrid work by showing that employees’ experiences are organized into distinct configurations of work-related resources rather than varying along single dimensions. By identifying qualitatively different profiles of hybrid work quality, the findings demonstrate that hybrid work is inherently heterogeneous and that this heterogeneity is systematically associated with different patterns of perceived team functioning. These results extend the Quality of Telework framework by conceptualizing it as a configurational model in which multiple resources jointly shape experiential patterns. In doing so, the study highlights the importance of examining how combinations of conditions, rather than isolated factors, are linked to team-related perceptions and outcomes in contemporary work settings. More broadly, the findings support the relevance of personoriented approaches in organizational research, showing how configurations of perceived work conditions are associated with qualitatively distinct patterns of experience. Adopting such an approach allows for a more nuanced understanding of complex work phenomena and provides a useful framework for capturing heterogeneity in hybrid work contexts.

## Data Availability

The datasets used and analysed during the current study are available from the corresponding author on reasonable request.

## Action editor

Lars-Gunnar Lundh served as action editor for this article.

## Open access

This article is distributed under the terms of the Creative Commons Attribution 4.0 International License (http://creativecommons.org/licenses/by/4.0/), which permits unrestricted use, distribution, and reproduction in any medium, provided you give appropriate credit to the original author(s) and the source, provide a link to the Creative Commons license, and indicate if changes were made.
